# Development of a Quality Improvement/Patient Safety Curriculum to Increase Emergency Medicine Resident Scholarly Activity

**DOI:** 10.51894/001c.5044

**Published:** 2016-10-24

**Authors:** Nik Butki, Martina Ghiardi, William D. Corser

**Affiliations:** 1 McLaren Oakland Hospital Emergency Medicine Residency Program; 2 Michigan State University College of Osteopathic Medicine, Statewide Campus System

**Keywords:** scholarly activity, patient safety, quality improvment

## Abstract

**CONTEXT:**

There currently is no standard method for teaching Quality Improvement/Patient Safety (QIPS) content to prepare resident physicians planning QIPS projects. As part of the 2015-2016 MSU Statewide Campus System *Teach for Quality* (Te4Q) learner cohort, the first two authors from the McLaren Oakland Hospital Emergency Medicine (EM) residency program developed a structured multi-phase QIPS curriculum. The curriculum was developed to help a cohort of seven second-year EM residents feel more confident to design and conduct their own QIPS projects.

**METHODS:**

After institutional review board project approval was obtained, the first two authors evaluated both the pre and post-curriculum confidence survey scores of enrolled EM residents during May, 2016 as part of their Te4Q program participation.

**RESULTS:**

Residents completed a 15-item QIPS confidence survey before and after completing the QIPS curriculum. The mean pre-curriculum score was 3.00 (SD 1.53) on a scale from 0 to 10, indicating that the average sample respondent felt a lower level of comfort concerning their ability to design and conduct a prospective QIPS project. The mean post-curriculum confidence score from residents increased to 6.71 (SD 1.25) on a 0 to 10 scale, over double an increase from the pre-workshop score on this item. Using a series of non-parametric Wilcoxon Matched Pairs Signed Rank Test procedures suitable for smaller samples, statistically significant increases in pre- to post-curriculum differences were shown for composite confidence scores (Z = 2.207, *p* = 0.027), as well as for five of the 12 individual confidence items (p-values ranged from 0.023 to 0.046).

**CONCLUSIONS:**

These initial results certainly indicate that a structured ongoing QIPS curriculum may have the potential to improve EM residents’ confidence levels to design and implement QIPS projects with faculty. The impact of these types of curricula for EM and other types of residents needs to be more rigorously examined in more tightly controlled GME settings with larger samples to gauge what types of resident learners will more likely benefit from such educational offerings across the nation.

## INTRODUCTION

A growing awareness of the importance of conducting Quality Improvement/Patient Safety (QIPS) projects in healthcare environments was emphasized in the 2000 Institute of Medicine publication *To Err is Human:* *Building a Safer Health System.*^1^ This group of healthcare experts cited evidence showing that hospital medical errors were associated with between 44,000 to 98,000 annual patient deaths. In a related move, the Accreditation Council for Graduate Medical Education (ACGME) has incorporated systems-based practice competencies that require residency programs across the nation teach residents “an awareness of and responsiveness to the larger context and system of health care, as well as the ability to call effectively on other resources in the system to provide optimal health care” and have residents “participate in identifying system errors and implementing potential systems solutions.”^2^

During recent years, the broader term “scholarly activity” also has been used by the ACGME in different accreditation documentation to describe both QIPS and research-oriented projects, as well as other types of professional development activities in graduate medical education (GME) settings.^3,4^

However, currently there is still no standard GME method to provide QIPS project content to residents preparing to plan and conduct such projects.^5–8^ In order to address this deficiency, the first two authors (comprised of one faculty [NB] and one senior resident [MG]) from the four-year McLaren Oakland Hospital Emergency Medicine residency program developed an EM-specific QIPS curriculum. These authors developed the curriculum as members of the 2015-2016 *Teach for Quality* (Te4Q) program^9^ offered by the Michigan State University (MSU) Statewide Campus System in East Lansing, Michigan.^10^

Originally, the Association for American Medical Colleges had developed the Te4Q program to train university-based faculty teams in single institutions to provide residents with key QIPS content and skills. This was the first time that the Te4Q program had been offered to a statewide network of community-based attending faculty members. Each of the 19 participants from 13 different residency settings was affiliated with one of the 37 healthcare systems served by the MSU Statewide Campus System consortium. The elements of this modified Te4Q program for the cohort of community-based learners have already been described in another paper by the third author and colleagues.^11^

## PROJECT PURPOSE

The overall purpose of this project was to develop a setting and clinical specialty-specific curriculum to train a cohort of second-year EM resident physicians at McLaren Oakland Hospital in Pontiac, MI. The desired outcome of the curriculum was to help each resident develop an individual QIPS project by the end of their second residency year (i.e. June, 2016). This article will review the McLaren Oakland authors’ curriculum development process, report their promising pre-post workshop evaluation results, and discuss potential modifications for the implementation of similar training currcula in other GME settings. The first two authors (NB & MG) had earlier identified that the manner in which QIPS content had been routinely delivered to all earlier-year EM residents in this setting had been both inconsistent and unevaluated. Although the QIPS curriculum described in this paper had already been incorporated into the GME offerings assigned for each second-year EM residents, residents’ choice to complete pre- and post-curriculum surveys was optional.

## EM CURRICULUM DEVELOPMENT

The overall **goals** of the QIPS curriculum were to: 1. improve EM residents’ confidence in using key QIPS project-related skills to develop an initial project and utilize these skills in their future EM practices after graduation; 2. meet the scholarly activity ACGME accreditation metrics and increase the number of dissemination products of EM residents and faculty within this Michigan residency program; and 3. eventually improve the overall quality and safety of care delivered to over 35,000 annual patients in the McLaren Oakland Emergency Department.

The specific **objectives** of the customized curriculum were to: 1. train a group of EM residents to: a) identify feasible QIPS project topics, b) design their respective projects using the *Plan-Do-Study-Act* (PDSA) format,^12^ c) conduct their project, and d) measure project outcomes. The McLaren Oakland faculty authors leaders developed the curriculum based on Te4Q modules and the QIPS curricular development work by Dr. Brian Wong and colleagues.^13^

The curriculum included five overall activities:

**1. QIPS Content Workshop.** This first workshop was designed and presented in July, 2015 by the McLaren Oakland authors in consultation with onsite colleagues and the Statewide Campus System project “coaches.” The initial four-hour workshop and the other curricular activities were incorporated into the pre-existing educational activities of the residency program. This first workshop introduced the basic concepts of QIPS, provided examples of QIPS projects appropriate for EM practice settings, and worked through a “cause and effect” or “fishbone” diagram with residents. The faculty and coaches also later demonstrated use of the PDSA cycle for proposed projects, and helped residents identify potential project measures and gain an appreciation for the frequently necessary processes for institutional review board (IRB) approval.

**2. Resident-Faculty Think Tank Planning Sessions**. During July and August, 2015, (i.e., two and four week intervals after the initial QIPS workshop) residents met with faculty again to discuss and evaluate each resident’s project idea(s). During the sessions residents each presented the perceived merit of their developing project designs, described the main components of their projects, as well as their initial plans for evaluating and disseminating project outcomes. Participants chose their “final” project topic following this think tank session and were paired with an attending faculty physician based on the attending’s areas of expertise. The third session, conducted during the fourth post-workshop week, included support from Statewide Campus System QIPS specialists, who helped participants identify project-related resources, provided topic-pertinent journal articles, and provided them with individualized written project feedback.

**3. PDSA Implementation.** During the next six months the residents met/communicated virtually with one another and/or in small groups with the McLaren Oakland authors, assigned attending faculty and two Statewide Campus System coaches to monitor the progress of their projects, troubleshoot identified barriers, modify projects if needed, and discuss future dissemination venues (e.g., poster sessions, conferences, publication in journals) for their completed project results.

**4. Follow-Up Project Workshop**. This two-hour follow-up workshop took place during January 2016, six months after the initial content workshop, and was designed and facilitated by the McLaren Oakland authors to assess participant progress and assist with PDSA-cycle issues.

**5. Final Project Review**. This final phase of the total curriculum will be completed in August 2016, and will entail review of residents’ final QIPS project design.

## METHOD

After McLaren Oakland IRB project approval was obtained, the authors evaluated the pre- and post-curriculum survey data from the seven enrolled EM residents for the McLaren Oakland authors to fulfill the requirements of their own Te4Q program participation. This report provides the analytic results of these mid-, pre- and post-curriculum surveys.

*SAMPLE*: The 2015-2016 class of second-year emergency medicine residents (n = 7) at McLaren Oakland Hospital participated and completed both pre- and post-curriculum confidence level surveys.

**Figure 1.**
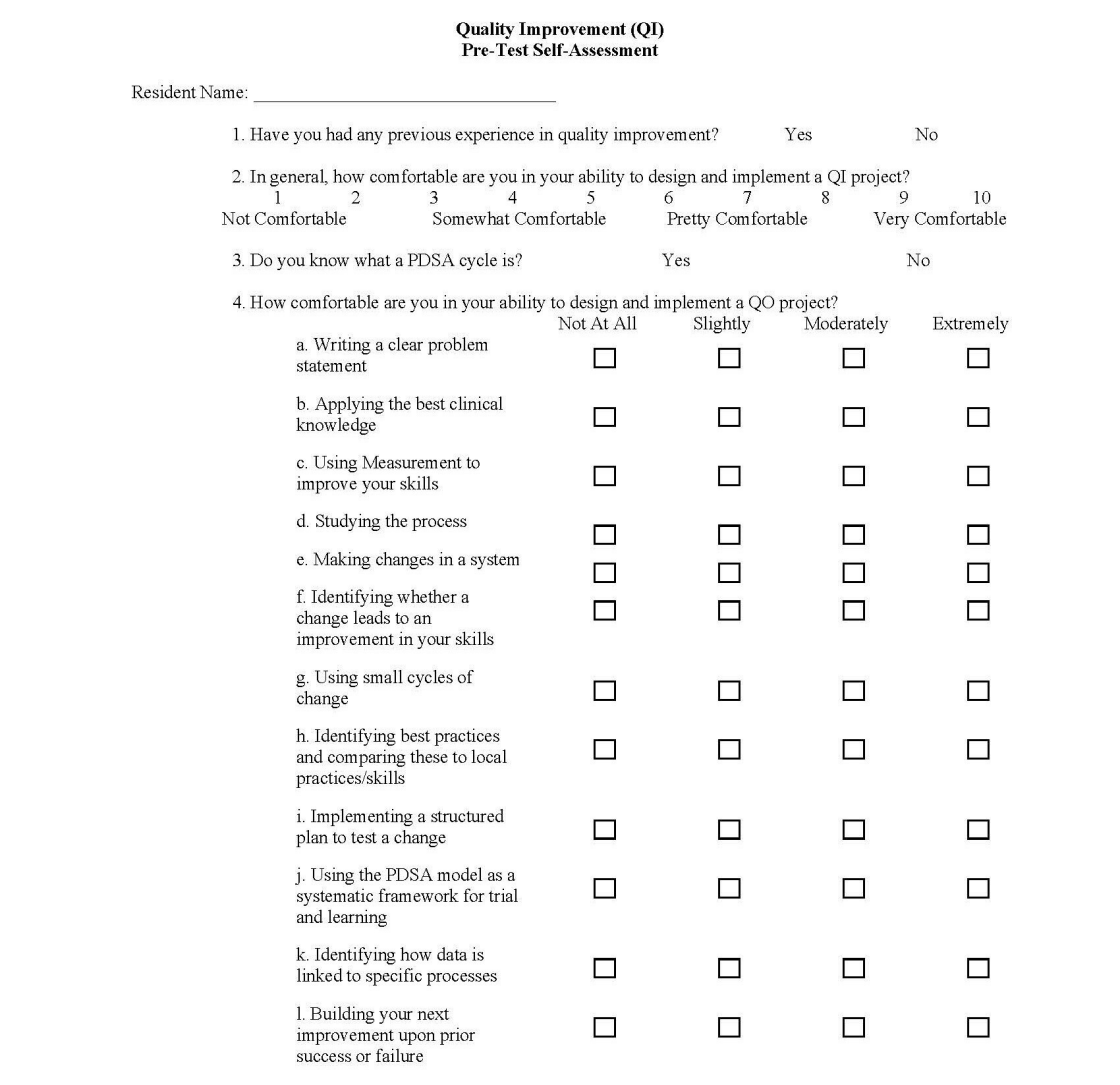
Pre- and Post-Workshop Survey Instrument Adapted from: Miller K, Knight L. “Quality Improvement (QI) to the Max. A study on the effects of a structured QI curriculum on resident confidence in using QI methods.” (2014). Department of Pediatrics, University of South Carolina School of Medicine/Palmetto Health Children’s Hospital, Columbia, SC.

*MEASURES AND TIMEFRAME*: Prior to the first QIPS Content Workshop, residents completed a 15-item *Resident Confidence in using Quality Improvement Methods* (RCQIM)^14^ survey questionnaire. This survey, partially derived from the validated Oyler et al. 2008 tool,^15^ asked respondents to rate on an 11-point Likert scale, from 0=*Not Comfortable* to 10=*Very Comfortable*, their overall personal comfort level *to design and* *implement* a prospective QIPS project. Respondents also were asked to rate their confidence concerning 12 specific aspects of developing and conducting a QIPS project on a different 4-point Likert scale, from 0=*Not at all Comfortable* to 3=*Extremely Comfortable* (see Figure 1).

During the follow-up project workshop, residents were again asked to complete an identical copy of the RCQIM. To help inform future curricular refinements, the residents also were invited to complete an additional on-line seven-item Survey Monkey^16^ questionnaire to evaluate their satisfaction with the QIPS curriculum. These items included:

Were you satisfied with the course content, neither satisfied nor dissatisfied with it, or dissatisfied with it?

Were you satisfied with your instructors’ teaching, neither satisfied nor dissatisfied with it, or dissatisfied with it?

How well-organized was this course?

How easy is it to get the resources you need to conduct your research at the hospital?

What suggestions do you have for improving this QIPS curriculum program?

*What were your least favorite experiences during the QIPS curriculum program?* and

Please provide any additional concerns, improvements, or comments you have regarding the course.

*DATA ANALYSIS*: Preliminary descriptive statistical and graphs confirmed that the distributions of RCQIM composite (i.e., 0 to 10) and individual item (0 to 3) scores were non-parametric (i.e., not normally distributed), as might be expected from a smaller sample in a single residency program. In response, a series of non-parametric Wilcoxon Matched Pairs Signed Rank Test procedures^17^ were conducted by the third author using *SPSS Version 22* analytic software.^18^. These tests were completed to compare differences in composite and individual item and composite pre- and post-curriculum resident confidence scores for potential statistical significance, observing a 0.05 p-value significance level.

## RESULTS

### Descriptive Statistics

*PRE-CURRUCULUM SURVEYS:* A total convenience sample of seven EM residents completed the 15-item RCQIM confidence survey both before and after the QIPS content workshop. Respondents also were asked to report whether they possessed any prior experience with any QIPS project(s). Only one respondent (14.3% of sample) indicated they had completed any QIPS project experiences. With regard to the RCQIM overall confidence item, the sample mean was 3.00 (SD 1.528) (on the 0 to 10 scale), indicating that the average respondent felt a lower level of comfort concerning their ability to identify, design, and/or conduct a QIPS project (see Table 1).

**Table 1:** **Descriptive Statistics of RCQIM Scores** (N = 7 Second-Year Emergency Medicine Residents)

	Pre-Curriculum Mean (SD) (range)	Post-Curriculum Mean (SD) (range)
**I. Overall Comfort Level to Design and Implement a QIPS Project**	**3.00**	**6.71**
(range 0 to 10)	(1.528) (1-5)	(1.254) (5-9)
**II. Composite Confidence Scores**	**16.57**	**23.86**
(range 0 to 36)	(4.198) (12-25)	(4.741) (17-30)
**III. Individual Confidence Scores** (range 0 -3)		
1. “Writing a Clear Aim Statement"	**1.14**	**2.29**
	(0.378) (1-2)	(0.756) (1-3)
2. “Apply Best Professional Knowledge”	**1.57**	**2.29**
	(0.535) (1-2)	(0.756) (1-3)
3. “Use Measurement to Improve Skills”	**1.57**	**1.71**
	(0.535) (1-2)	(0.488) (1-2)
4. “Studying Selected Process”	**1.57**	**2.00**
	(0.535) (1-2)	(0.816) (1-3)
5. “Make Changes in a System”	**1.29**	**1.86**
	(0.488) (1-2)	(0.378) (1-2)
6. “Identify whether Change led to Personal Skills Improvement”	**1.71**	**2.00**
	(0.690) (1-3)	(0.000) (2-2)
7. “Using Small Cycles of Change”	**1.86**	**2.00**
	(0.690) (1-3)	(0.577) (1-3)
8. “Identify Best Practices and Compare to Local Practices/Skills”	**1.00**	**2.14**
	(0.577) (0-2)	(0.378) (2-3)
9. “Implement a Structured Plan to Test Change”	**1.14**	**2.00**
	(0.690) (0-2)	(0.577) (1-3)
10. “Use PDSA Model as Systematic Framework”	**1.43**	**1.71**
	(0.535) (1-2)	(0.756) (1-3)
11. “Identifying how Data is Linked to Specific Process”	**1.14**	**1.71**
	(0.378) (1-2)	(0.488) (1-2)
12. “Building Next Improvement upon Prior Success/Failure”	**1.29**	**2.14**
	(0.756) (1-3)	(0.690) (1-3)

The composite pre-curriculum RCQIM confidence scores obtained from the 12 individual survey items also were quite low, averaging 16.57 (SD 4.20) on a possible scale from 0 to 36, but ranged from 12 to 25 per individual respondent. From these individual RCQIM items, the lowest average score was obtained for the item concerning *Identifying best practices and comparing these to local practices/skills* (mean 1.00, SD 0.577, on a 0 to 3 scale). Several items shared the same mean (1.114): *Ability to write a clear AIM statement*; *Developing* *a structured plan to test a proposed change*; and *Identifying how data is linked to specific QIPS processes*. The highest average pre-curriculum response was obtained for *Using small cycles of change*, which still only averaged 1.86 (SD 0.690) on a 0 to 3 scale. Additional higher than average items were *Making changes in a system* and *Identifying if a change leads to improvement*, both with Means of 1.78, SD 0.833 (see Tables 1 and 2).

**Table 2.** **Pre and Post-Curriculum RCQIM Scores** (N = 7 Second Year Emergency Medicine Residents *

	Pre-Curriculum Mean Score	Post-Curriculum Mean Score	Difference	Z Score	Significance
**I. Overall Comfort Level to Design and Implement a QIPS Project** (range 0 to 10)	3.00	6.71	+ 3.71	2.214	**0.027**
**II. Composite RCQIM Scores** (range 0 to 36)	16.57	23.86	+ 7.29	2.207	**0.027**
**III. Individual Confidence Item Scores** (range 0 -3)					
1. “Writing a Clear Aim Statement”	1.14	2.29	+ 1.15	2.070	**0.038**
2. “Apply Best Professional Knowledge”	1.57	2.29	+ 0.72	1.518	0.129
3. “Use Measurement to Improve Skills”	1.57	1.71	+ 0.15	0.577	0.564
4. “Studying Selected Process”	1.57	2.00	+ 0.43	1.414	0.157
5. “Make Changes in a System”	1.29	1.86	+ 0.57	2.000	**0.046**
6. “Identify whether Change led to Personal Skills Improvement”	1.71	2.00	+ 0.29	1.000	0.317
7. “Using Small Cycles of Change”	1.86	2.00	+ 0.14	0.577	0.564
8. “Identify Best Practices and Compare to Local Practices/Skills”	1.00	2.14	+ 1.14	2.271	**0.023**
9. “Implement a Structured Plan to Test Change”	1.14	2.00	+ 0.86	1.890	0.059
10. “Use PDSA Model as Systematic Framework”	1.43	1.71	+ 0.28	0.707	0.480
11. “Identifying how Data is Linked to Specific Process”	1.14	1.71	+ 0.57	2.000	**0.046**
12. “Building Next Improvement upon Prior Success/Failure”	1.29	2.14	+ 0.85	2,121	**0.034**

* Series of Wilcoxon Matched Pairs Signed-Rank Tests

Statistically Significant Differences at Alpha of less than 0.05 are listed in **Bold** font

*POST-CURRICULUM SURVEYS:* Overall post-curriculum (i.e., six months after the QIPS content workshop) confidence responses from residents averaged 6.71 (SD 1.254) (on a 0 to 10 scale), over double the mean of 3.00 for this pre-workshop survey item. This improvement suggests the average respondent became significantly more confident in designing and conducting a future selected QIPS project. The composite post-curriculum RCQIM scores also increased considerably, averaging 23.87 (SD 4.64) out of possible scores from 0 to 36, an increase of 7.3 points, but still ranged widely from 17 to 30. In terms of RCQIM composite score improvements, only one resident reported feeling no change in their confidence levels, with the remainder of respondents increasing from 4 to 18 points in their composite confidence scores (see Figure 3).

**Figure 2.**
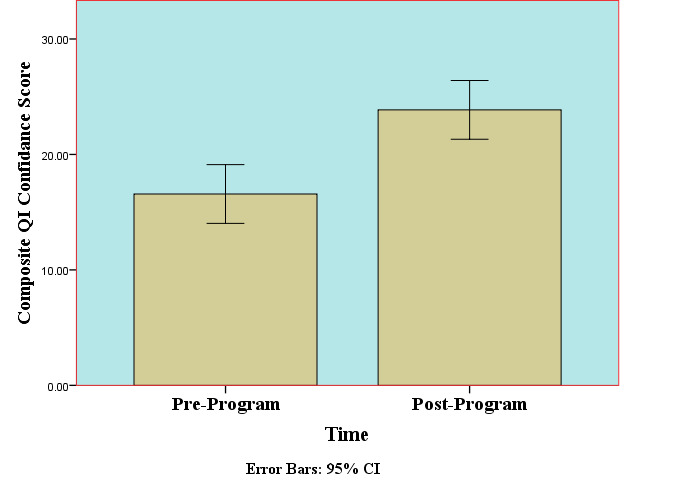
Comparisons of Pre- and Post-Curriculum QIPS Project Composite Confidence Scores (N=7 Emergency Medicine Residents)

**Figure 3.**
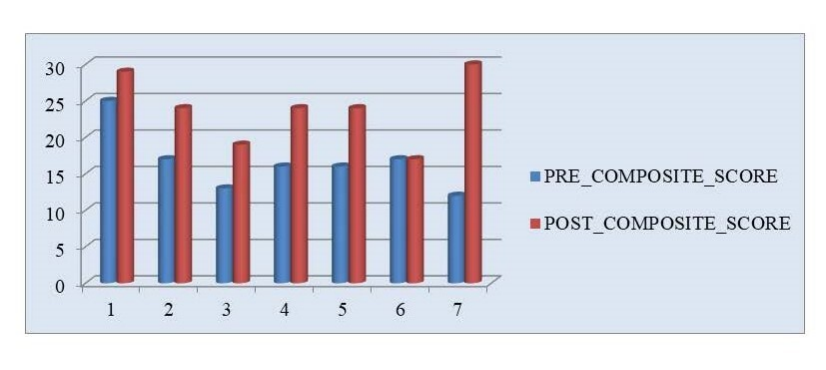
Pre- to Post-Workshop QIPS Project Confidence Score Changes (N = 7 Emergency Medicine Residents)

For the individual post-curriculum RCQIM survey items, the lowest average scores were obtained for the following two items: *Using a PDSA model as a systematic framework* and *Identifying how* *data is linked to a specific process* (both with a mean of 1.71; SD 0.756 and 0.488, respectively). The highest post-workshop survey item responses were for *Writing a clear problem statement* and *Applying best* *professional knowledge*, both with a mean 2.29 and SD of 0.756 on a possible 0 to 3 scale (see Table 1).

*MID-CURRICULUM SURVEY COMMENTS:* To evaluate how their initial QIPS curriculum had been received by the EM residents, an online SurveyMonkey survey was administered, with results summarized below.

1. *Satisfaction with Curriculum Content* (5 responses): Responses ranged from *Extremely Satisfied* to *Neither Satisfied nor Dissatisfied*.
2. *Satisfaction with Instructors’ Teaching* (5 responses): Responses ranged from *Extremely Satisfied* to *Neither Satisfied nor Dissatisfied*.
3. *Organization of Course* (5 responses): Responses ranged from *Extremely well-organized* to *Moderately well-organized*.
4. *Ease of Resource Obtainment* (5 responses): Responses ranged from *Extremely easy* to *Moderately* *easy*.
5. *Suggestions for improving program* (2 responses): a. *Continue to have check-ins/deadlines*, b. *Easy to* *let QI projects get put on back burner*, c. *Nothing*, and d. *I think facilitators did a great job keeping* *residents informed*.
6. *Least favorite experiences* (3 responses): a. *Coming up with an idea*, and b. *Having to continually* *review others’ projects*.

### Inferential Data Analyses

Using *SPSS Version 22* software, pre- and post-workshop RCQIM composite comfort ratings levels were shown to have increased significantly (Z = 2.207, p = 0.027), as did five of the 12 individual confidence items (these significant p-values ranged from 0.023 to 0.046; see Table 2). These test statistics were obtained using a series of Wilcoxon Matched Pairs Signed Rank Test non-parametric procedures that are particularly suitable for smaller samples that are not normally distributed.^17^

### Limitations

These initial project results should be viewed within the context of several clear limitations. The results are based on an extremely small convenience sample of EM residents in a single mid-Michigan EM residency program setting. The project was very likely underpowered to detect meaningful sample subgroup differences (e.g., male residents versus female residents) relative to pre- and post-curriculum score differences that may have been detected with a larger multi-program sample. It also should be acknowledged that measured increases in residents’ QIPS project confidence scores may have been skewed by some degree of *Hawthorne/observer effect*, since respondents obviously knew that they (and their scores) were being watched by McLaren Oakland faculty.

## CONCLUSIONS

These initial findings clearly suggest that a structured multi-phase QIPS educational curriculum has the potential to improve EM residents’ confidence levels to design and implement selected QIPS projects with faculty. At this point, the authors are generally satisfied with the post-curriculum confidence level increases measured during this study. Each resident project has obtained IRB approval, although the authors have concluded that it is likely still too early to evaluate the final curriculum outcomes until all projects have been completed. The impact of these types of curricula for EM and other residents needs to be more rigorously examined in more tightly controlled GME settings with larger samples to tease out what specific types of curricular activities might prove to be most effective for diverse resident learners across the nation.

In hindsight, the authors plan to make the following adjustments in their next curriculum offering:

1. More clearly delivering integrated workshop activities geared to the survey items of selected evaluation measure(s);
2. Purposefully working to address system-level barriers for residents during the earlier months of their project. The McLaren Oakland authors and others have concluded that such barriers (e.g., unclear processes related to access of project data, difficulty obtaining project related resources) can delay many residents’ progress with their projects;^11^,^19–22^
3. The number of QIPS workshops should probably be increased to three sessions from the original two, with each session two to four hours in length. The authors concluded that more time was required to help residents develop project planning skills such as working with: a) a statistician to develop appropriate evaluation and analytic methods; b) experienced project design or QIPS department personnel to develop feasible projects; and c) medical librarians to identify pertinent literature related to best practices in their selected area.

Anecdotally, the time spent assisting residents to clearly formulate a PDSA statement, and providing them ready access to consultation with campus-based QIPS experts, seemed to play a significant role in improving residents’ project-related confidence.

The measured improvements seen during this project suggest that the targeted design and delivery of such QIPS educational curricula in similar community-based GME settings is warranted. Ideally, similar QIPS project curricula will be developed in other settings as GME officials sort out the best means of effectively meeting more rigorous ACGME accreditation standards in residency programs across the nation.

## Conflict of Interest

The authors declare no conflict of interest.
